# A clustering metaheuristic for large orienteering problems

**DOI:** 10.1371/journal.pone.0271751

**Published:** 2022-07-22

**Authors:** Almiqdad Elzein, Gianni A. Di Caro

**Affiliations:** Carnegie Mellon University in Qatar, Doha, Qatar; National Taiwan University of Science and Technology, TAIWAN

## Abstract

The Orienteering Problem is a routing problem that commonly appears in mapping, transportation, distribution, and scheduling scenarios. The Orienteering Problem is a challenging NP-hard problem, such that obtaining optimal or near optimal solutions usually requires a significant amount of computation for large and even moderately large instances. As a result, existing algorithms cannot effectively be utilized for solving large Orienteering Problems in an online setting, which is often required in real-world applications where a plan has to be iteratively computed. For instance, a planner might need to adapt to changes in the scenario or in the environment (e.g., this is common in goods delivery, as well as in mobile robotic scenarios for coverage, monitoring, and surveillance). Motivated by these limitations, we propose a multi-stage clustering-based metaheuristic for tackling large Orienteering Problems in an effective, strategically controlled amount of computation time. The metaheuristic starts by decomposing the problem into smaller, independent sub-problems that are efficiently solved using an algorithm of choice, sequentially or in parallel. The obtained solutions are then merged to form a solution for the original problem, and then further optimized and processed to ensure feasibility. The metaheuristic aims to dramatically improve the computation time of a given Orienteering Problem algorithm without a significant decrease in the solution quality of that algorithm, especially for large Orienteering Problems. We have validated the effectiveness of the proposed metaheuristic design through a set of computational experiments. In particular, using a state-of-the-art heuristic and an exact algorithm, we have shown that it is significantly beneficial to use the Orienteering Problem algorithm plugged into our metaheuristic, as opposed to using it as a standalone algorithm. This was found to be especially true for large problems. As a result, large instances of Orienteering Problems can be effectively tackled within reasonable time bounds even for online application scenarios.

## 1 Introduction

The *Orienteering Problem* (OP) [1] is a form of routing problem with profits [2, 3]. In its basic, single-agent formulation, given a set of candidate sites, a cost for going from one site to another, and a numeric reward provided by each site, the goal is to select a subset of the sites and define the order in which to visit them so that the total collected reward is maximized. Additionally, a given cost budget (e.g., a traveling time) must not be exceeded. When the scenario features multiple agents to plan for, the OP becomes a *Team Orienteering Problem* (TOP) [4, 5], where each agent is subject to a similar cost budget and the goal is to maximize the cumulative reward. OPs and TOPs naturally arise in areas such as transportation and logistics, where, for instance, sites usually correspond to customers and agents correspond to vehicles used for the delivery of goods under different constraints [6–9]. Orienteering models have however been used in a much wider range of application scenarios, including recommender systems for the design of tourist trips, allocation of resources in civilian and military operations, emergency response, search and rescue, and task outsourcing using a crowdsourcing mechanism (see [10] for a recent and extensive review of applications of OP models).

Outside of typical operations research applications, OPs are also widely employed in scenarios featuring the use of *mobile robotic agents*. In particular, in scenarios of *surveillance* [11] and *monitoring* [12], OPs are used as optimization models for target selection and path planning. These scenarios are part of the more general class of so-called *informative path planning* (IPP) problems. Such problems consist of a robot or a team of robots having to select a number of sites to travel through based on an estimated measure of value or *information* associated with each site and subject to a limited time budget [13–15]. In IPP problem scenarios, it is often a requirement for the system to perform *online* path planning and replanning, to effectively account for progressively gathered evidence data (e.g., [15, 16]). Moreover, in IPP scenarios such as environmental monitoring, the set of sites which is considered in the OP model is usually obtained from the discretization of a continuous environment of reference. Clearly, the smaller the cardinality of the discretization, the potentially larger the loss in terms of total information that can be acquired. In other words, a possibly large set of sites to choose out of would be necessary to ensure an optimized result. Unfortunately, tackling a large OP presents major computational challenges.

The class of orienteering problems is in fact notoriously NP-hard [17] and presents severe computational challenges. For the OP, which is the focus of this work, a number of constant-factor approximation algorithms have been developed for special cases of the problem [18]. In general, even when using state-of-the-art heuristics [19], the time for tackling a (single-agent) OP with a few thousands candidate sites can grow up from several minutes to hours. This sets a barrier for efficient solving over *large domains*. This limitation is particularly important when decisions must be taken *online*, such as in typical robotic or logistics scenarios where the operational conditions are subject to frequent changes.

In this work we address the challenge of efficiently tackling *large OP instances* by proposing a novel multi-stage *metaheuristic*. This metaheuristic allows us to strategically balance solution quality and computation time to achieve effective and efficient online computations (e.g., on board of robots). Our metaheuristic is based on the automatic partitioning of a potentially large set of candidate sites into a much smaller set of *clusters* where a user-selected solver is employed to find an optimal or near-optimal solution in a small, bounded time. Following a staged approach, the solutions across the clusters are then suitably *merged* and the overall path is both *repaired* to ensure compliance with problem’s constraints and *locally optimized* to provide a final high-quality solution path. In practice, the metaheuristic can dramatically improve the time performance of a selected reference solver at the expense of very little or no loss regarding solution quality. Over a set of benchmark instances of different sizes, we have empirically assessed the efficacy of the metaheuristic considering one state-of-the-art heuristic solver based on evolutionary computation, *EA4OP* [19], and one state-of-the-art exact solver, Gurobi [20].

The metaheuristic goes through *multiple stages* of optimization that can be independently tuned and is inherently *parallelizable* using multi-core architectures. It also depends on a small number of parameters that can be strategically set or are internally estimated. Finally, our metaheuristic allows us to *plug-in any desired solver* (either exact or heuristic) to improve its performance.

**The main contribution of this work** is a novel multi-staged clustering-based metaheuristic that can be used with *any* OP algorithm and that can significantly enhance the computation time of that algorithm at the expenses of little (or no) loss in the quality of the final solution. The metaheuristic is aimed at greatly increasing the scale of OPs that can be effectively tackled when only a relatively tight time budget is available for computations. The substantial gain in computation time, coupled with the limited loss in solution quality (or even an improvement is some cases), allows for the effective use of the metaheuristic in online OP applications, particularly in those featuring a large numbers of candidate points (e.g., IPP problems in robotics for environmental monitoring and surveying, logistics and transportation scenarios in a dynamic environments). By exploiting its multi-stage organization and the data decomposition approach, the metaheuristic gives to the user a full strategic control for allocating computational resources and naturally allows users to exploit parallelism.

**The paper is organized as follows**. In Section 2, we review related work targeting solutions approaches that can tackle large orienteering problems, as well as the use of orienteering models in robotics, where typical online operations require fast computations. The clustering-based metaheuristic is presented in Section 3, where the different stages of processing are discussed in separated subsections. In Section 4, we report the results for the empirical validation of the proposed approach using two state-of-the-art solver, one heuristic and one exact. Finally, in Section 5, we draw final conclusions and outline future work.

## 2 Related works

### 2.1 Approaches for large orienteering problems and use of partitioning methods

There exist a number of different solutions approaches for OPs (e.g., see [1] for an overview), accounting for a diversity of formulations, objectives, and constraints. Here, we review selected works specifically addressing the suitability of an approach for efficiently tackling large OP instances. We also specifically review existing work that is based on or relies on data partitioning, similar to our data decomposition using clustering. Note that the qualitative assessment of the performance of each algorithm is done referring to the benchmark set and the computational experiments of the EA4OP paper [19], which we use as reference in the experiments reported in Sec. 4.

Silberholz et al. [21] propose a Two-Parameter Iterative Algorithm (2-P IA) for the *General Orienteering Problem* (GOP), which is is an OP where each candidate point is assigned a number of scores for different attributes and the overall function to optimize is a function of these attribute scores. The results of OPs solved using the 2-P IA heuristic show that, while the algorithm is very competitive in regards to solution quality, its computation time does grow drastically, and impractically, for large instances with one thousand points and more, which is a common pattern for existing heuristic approaches.

Remarkable examples of heuristics for OPs include GRASP with Path Relinking (GRASP-PR) [22] and the evolutionary algorithm EA4OP proposed by Kobeaga et al. [19]. Similar to 2-P IA, these heuristics are able to produce near-optimal solutions, but with a computation time that increases significantly for large instances, which is especially the case for GRASP-PR. The incorporation of any of these algorithms into our metaheuristic is expected to result in a large decrease in computational time accompanied by only a small decrease in solution quality. We have assessed this for the case of EA4OP (given that the code was publicly available).

Santini [23] proposed a heuristic based on the Adaptive Linear Neighbourhood Search (ALNS) algorithm. The heuristic starts off with an initial solution and iteratively destroys and repairs the solutions using different destroy methods, such as random and cluster remove methods, and different repair methods, such as a greedy and cluster repair methods. A local search method is optionally executed on the solution after it is destroyed and repaired to further improve it. The best feasible solution is then chosen. The reported experimental results for this heuristic show that it outperforms other heuristics, including the EA4OP that we use in this work, in many instances, but it has a worse time efficiency than EA4OP for large instances. High-quality yet relatively inefficient heuristics such as the one in [23] are suitable candidates to plug into our clustering metaheuristic. Note that we have chosen to use EA4OP heuristic as a reference here given the availability of the source code and its relatively simple(r) structure.

Gavalas et al. [24] have proposed two cluster-based improvements to Iterative Local Search in team-orienteering problems. The two methods proposed are called: Randomized Cluster Ratio (RCR) and Cluster Search Cluster Routes (CSCR). CSCR enforces that all nodes in a cluster are visited before moving to the next cluster. One the other hand, RCR makes it such that it is preferable to stay within one cluster until all of its nodes are visited. In principle, these heuristic for local serach could be incorporated into out metaheuristic to possibly boost the local search phase, at the expenses of extra computational costs.

Sylejmani et al. [25] consider a scenario where paths for a group of tourists have to be obtained and model this problem as a TOP. In this instance, however, tourists have heterogeneous preferences for the available tourist sites. Additionally, a tourist’s utility from visiting a site depends on who are the specific tourists visiting that site at the same time, based on a metric of social relations. The authors propose to cluster the tourists based on their closeness to each other, where closeness is measured as the Euclidean distance in a 4-dimensional space of different attributes. The members of each cluster are assigned the route of their cluster’s centroid. This approach departs from the scenarios that we are focusing on since it considers a specific class of TOP scenarios.

Archetti et. al. [26] formulate and propose a heuristic solution for the *Set Orienteering Problem* (SOP), a cluster-based generalization of the OP. In a SOP, candidate points are grouped into clusters and each cluster has a utility score associated with it. The utility of a cluster is gained by the orienteering agent if it visits at least one candidate point in that cluster. The proposed heuristic solution approach is based on Tabu Search [27]. Again, while the proposed solution is interesting in terms of using a clustering approach, it departs from the scenarios that we consider here since in this case it is enough to visit one point to clear one cluster.

On the side of *exact algorithms* for the OP, one reference is the Branch-and-Cut (B&C) algorithm proposed by Fischetti et al. [28]. From results reported in [19], we can see that (as expected) the algorithm’s computation time explodes for larger OP instances. The popular Gurobi solver offers a flexible standard interface for tackling OPs. In general, all the exact algorithms get slow when confronting a large instance, but are indeed relatively fast on solving small instances. In our metaheuristic we aim to exploit this fact, by automatically decomposing the input problem into small sub-problems that can be quickly solved to optimality by standard / optimized exact solvers. In this way we can reuse what is readily available, making it able to scale up its applicability to large(r) problems.

The idea of using *clustering* in the input space or, more generally, *data partitioning* has been applied to different types of routing problems, including different versions of the *Travelling Salesman Problem* (TSP) and of the OP, both for single and multi-agent cases. It is worth noticing that the OP is related to the *Selective TSP* [29].

In the case of the *Clustered Travelling Salesman Problem* (CTSP) [30], the notion of clustering is part of the problem definition itself. In an *N*-city CTSP, not only should each city point be visited exactly once, but subsets (clusters) of these cities must be visited contiguously. In a recent paper, Anaya et al. [31] proposed a solving the CTSP is by breaking up a TSP into smaller independent TSPs. The proposed solution utilizes a combination and modification of the heuristic NEH [32] and of the metaheuristic Multi Restart Iterated Local Search (MRSILS) [33]. After each sub-problem is solved, the solutions are joined with the aim of minimizing the total cost of the path. In [31], joining sub-solutions into one final solution is done by connecting each sub-solution to the closest sub-solution available. In our metaheuristic, we follow a similar approach, but instead of using a greedy solution-connecting procedure, we join sub-solutions through a TSP procedure that is better able to minimize the path cost of the overall solution and ensure overall feasibility. Moreover, we set the number of sub-problems and identify them in a fully automatic way (through K-medians clustering).

Another type of Clustered Traveling Salesman problem is the *General Traveling Salesman Problem* (GTSP) where the salesman is presented with a set of clusters and the goal is to visit exactly one vertex in each cluster. Karapetyan et al. [34] proposed a Lin-Kernighan heuristic for the GTSP. GTSP is quite a different problem from ours.

Angelelli et al. [35] address a generalization of the OP, which they call the *Clustered Orienteering Problem* (COP). A COP consists of clusters of customers that an agent must visit. A pre-determined reward is obtained when the orienteering agent visits all costumers in a cluster. This paper proposes two solution approaches, an exact solution approach, which is a branch-and-cut algorithm, and a heuristic algorithm based on a tabu search scheme.

Decomposing a TSP into smaller TSPs, similarly to what we do for an OP, is proposed by Deng et al. [36–38]. In [36, 37], *K-means* [39] clustering is used. On the other hand, affinity propagation [40] is used in [38]. We prefer to use *K-medians* [41] over these two clustering methods because it is more robust to outliers, unlike K-means, and it allows us to determine the number of clusters, unlike affinity propagation. Note that our metaheuristic must be able to choose the number of clusters because it allows us to limit the computation time required to obtain a final solution. As we will see in following sections, we choose the number of clusters based on the time-efficiency of the algorithm used to find the paths inside the clusters. To join the cluster solution into a final solution, a nearest neighbor approach is used in [36] and a genetic algorithm in [37, 38].

Sodhi [42] proposes another clustering heuristic for solving TSPs in order to improve the Greedy and the Nearest Neighbor TSP heuristics. Here, the author utilizes the Density-Based Spatial Clustering of Applications with Noise (DBSCAN) to cluster the TSP nodes. When using the Greedy heuristic, a solution for each cluster is found and these solutions are then joined greedily. On the other hand, when using the Nearest Neighbor heuristic, a different procedure is followed: once a node is visited that is within a cluster, the nearest neighbors algorithm is restricted to nodes that are within that cluster until all nodes in that cluster are visited. In our work, we opt for using distance-based clustering instead of density-based clustering, precisely aiming to minimize the distance between members of the same cluster. In fact, having clusters in which the points are close to one another is preferable since it would result in cluster solutions with lower costs, which can allow us to visit more nodes, and therefore potentially gather higher rewards in the OP.

Finally, Gavalas et al. [24] use two cluster-based heuristic algorithms to organize the candidate sites into groups based on topological distance criteria and intelligently support the use of an Iterated Local Search. The nodes inside a cluster are explored first, and then the search sequentially moves across adjacent clusters. The algorithms are developed for the *Team Orienteering Problem with Time Windows* (TOPTW), which is among the most challenging classes of orienteering problems. Clustering is done based on K-means. Results are qualitatively good and computations are fast. However, only instances up to a maximum of 250 nodes are considered, which is reasonable considered the complexity of TOPTW. In our approach, apart from considering OP instead of TOPTW, a cluster is seen as a sub-problem that can be independently solved, with the solution pieces being merged in a final phase. Our approach allows us to exploit parallelism of computations and implement multiple levels of optimization.

The metaheuristic that we propose applies the concept of problem decomposition already found in the literature, however, it includes a few innovative design aspects, such as: the use of a (more) robust clustering methodology, the strategic division of the budget among the sub-problems based on estimated total reward and the capabilities of the solver at hand, a fully parallelizable way of solving the sub-problems, a general yet efficient methodology for joining the solutions of the sub-problems and adjusting the composite path to guarantee budget feasibility and maintain high solution quality.

### 2.2 Orienteering models for informative path planning problems

So far we have reviewed existing work from the Operations Research domain. However, as pointed out in the Introduction, orienteering models are also widely employed in *mobile robotics*, especially when tackling IPP problems. In these cases, the scenarios typically require setting up large OPs that have to be solved online and iteratively (e.g., for a path replanning triggered by newly acquired data and/or changes in the environment). In this context, the availability of orienteering heuristics that can handle large problems in a short, controlled computation time is essential to permit an extensive and efficient use of OP models. Indeed, the design of the metaheuristic presented in this paper has been precisely motivated by the need to solve online large IPP problems arising in the context of a research addressing the use of multi-robot systems for surveying and monitoring tasks in marine environments (research project *TARMEM: Teams of aquatic and aerial robots for marine environmental monitoring*, www.tarmem.org).

Unfortunately, in addition to the computational challenges, the use of standard orienteering formulations in IPP scenarios can suffer from one additional issue, which is related to the use of an *additive objective function* in the OP model. In fact, in typical IPP scenarios such as environmental surveying, the goal is to reduce uncertainty in the target regression model (i.e., the spatial map of the environmental attribute of interest) which is incrementally built using the measures sampled by the robot(s) at the selected locations. In such scenarios, the information provided by adjacent locations is usually *correlated* (e.g., water salinity won’t change dramatically from one point to its close neighbor points), such that the information provided by the measure at one location depends on the history of the other nearby sampled locations. In other words, the contribution of each measure cannot be summed up independently from the other measures. In these cases, using a standard OP formulation with a modular (additive) objective defines a model which results in an *approximation* of the original underlying problem, being based on the assumption of *independence* for the information provided by the individual sites.

A number of works have specifically addressed and incorporated the above notion of correlation in information, resulting in a departure from the basic orienteering model. Krause et al. [43] and Chekuri et al. [18] show that using an appropriate measure of information such as mutual information allows us to define efficient algorithms and theoretical bounds for the *submodular orienteering problem* [44] (where the objective function becomes submodular). In [45] submodularity is used in a more general multi-robot scenario where IPP is coupled with the notion of risky traversal of an edge to define a linearization procedure which leads to a greedy approximation algorithm with a constant-factor approximation guarantee. Yu et al. [12] assume correlation but do not assume submodularity. Instead, a general *correlated orienteering problem* is defined and formulated as a mixed integer quadratic programming, which is solved by a newly proposed algorithm. In other works [13–15], different optimization approaches have been used for tackling the IPP problem, always explicitly tackling into account the statistical correlation structure of the objective function.

The downside of these approaches accounting for a submodular objective is that the number of sites that are considered in practice for solving a correlated version of the OP is necessarily small, in the order of tens in [15, 46] and up to 150 in [12]. In other words, a large area to survey is drastically discretized into a small number of candidate locations, usually placed on a fixed regular grid, and the IPP is solved with respect to these points. Moreover, computations can still be relatively large, in the order of thousands of seconds, which is not suitable for an *online* setting.

These practical limitations suggest that an alternative way to tackle IPP problems can consist of using a modular approximation but coupled with the ability to solve large instances, with thousands of sites, quickly and using an iterative approach with a rolling horizon for frequent replanning. In other words, using large instances, a dense discretization can be realized, increasing the potential accuracy for selecting sites. At the same time, by exploiting the rapidity in tackling the instance, a continual replanning scheme can be in place, allowing us to account for the progressively gathered new evidence. Overall, these advantages can cancel out or reduce the impact of the approximation error introduced by the assumption of independence in the objective. An example of the effectiveness of such an approach is shown by Di Caro and Ziaullah Yousaf [16], which in turn further supports the need for fast heuristics capable of handling large OPs.

## 3 Clustering-based OP metaheuristic

**Fig 1:**
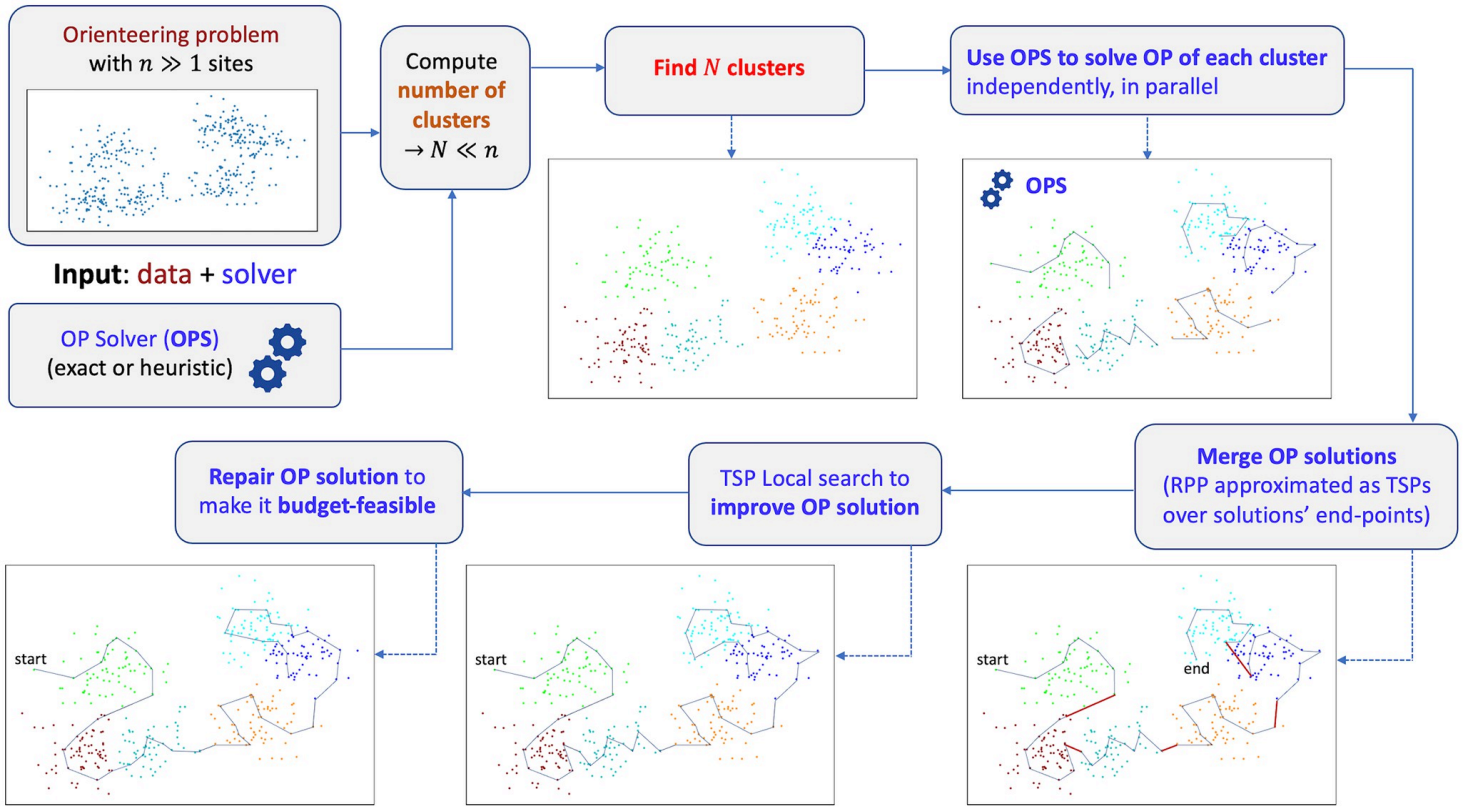
Clustering-based metaheuristic. Illustration of the logical organization and multi-staged workflow of the proposed clustering-based metaheuristic.

In this section we describe the proposed clustering-based metaheuristic aimed at efficiently and effectively dealing with large OP instances. The organization and the multi-staged working flow of the metaheuristic is illustrated in Fig 1. The metaheuristic takes as inputs the OP *instance data*, featuring a possibly large number *n* of candidate sites to select in the solution, and a *solver*, OPS, which can be either an exact or a heuristic OP solver. The idea is that, once used inside the metaheuristic, the OPS solver will be able to boost its own performance such that it will be able to tackle the large instance within a reasonably short amount of computation time, possibly allowing for an online use.

The OP instance is represented by an edge-weighted graph *G* = (*V*, *E*) with numerical profits / values on the nodes. Given a starting node *s* and a limited cost budget *B* for crossing the edges, the goal is to find a path from *s* to a terminal node *t* such that the total additive profit from the visited nodes is maximized while the budget *B* is not exceeded. Note that, without loss of generality, we are not necessarily assuming a pre-defined terminal node *t*. This does not change the range of applicability of the metaheuristic. At the same time, it reflects a possible use in a robotic scenario, where, for instance, an OP is iteratively computed over a rolling horizon, such that providing a target end-point might be an unnecessary constriction. In practice, we assume that the input data refer to a *rooted OP* [47], where the root might represent the current position of the agent, but no final site is assigned (i.e., it does not matter where the agent is located at the end of its journey). Note that such *start* position can be added to the data set as a location that provides zero reward. Note also that, in general, assuming a rooted OP is not restrictive. In fact, solution and approximation approaches for rooted OPs can be immediately adapted to unrooted versions, while the opposite is more problematic (e.g., see [48] for a more in-depth discussion).

We also make the assumption that the graph *G* is *connected*. This will be necessary to deal with merging the solutions from individual sub-problems. The assumption could be reasonably relaxed requiring, however, a more articulate strategy for merging, which we are not considering here.

The metaheuristic goes through *multiple stages* of processing that are detailed in the next subsections. Here we provide a step-by-step short summary:

1. **Definition of the cardinality of data partitioning**. As an initial step, a partitioning of the input data defining the given OP is performed with the aim of decomposing the initial, potentially large input space with *n* candidate data points into a set of *N* non-overlapping partitions. The value of *N* is selected to let each partition contain a number of data points much smaller than *n*. This is done to ensure that an OP defined over these points can be effectively tackled by the selected OPS within a strategically defined computation time *t*_*cluster*_. In other words, each partition defines an *orienteering sub-problem* which can be effectively solved in a short time.
2. **Partitioning in sub-problems by clustering**. The selected number *N* of partitions is then used as an input to a *clustering* algorithm that finds a good, balanced partitioning of the input data into *N* clusters adopting topological distance as grouping criterion. Each cluster defines an orienteering sub-problem *S*_*i*_, *i* = 1, …, *N*.
3. **Use OPS to independently solve the OP in each cluster**. Once we have defined the *N* sub-problems, each sub-problem *S*_*i*_, *i* = 1, …, *N* is solved *independently*, and possibly in *parallel*, by using the OPS. The size of each *S*_*i*_ is such that the OPS solver is adequate to solve the sub-problem within the strategically defined bounded time *t*_*cluster*_.
  Note that, in the cluster containing the *start* position, a rooted OP is solved, while in all the other clusters an *unrooted OP* is solved. If a final destination is given, this can easily be added. This is shown in Fig 1, where the ‘start’ site indicates where the agent initially is.
4. **Merge orienteering solutions from all sub-problems**. At this stage, we have *N* disjoint OP solutions as paths *P*^*i*^, *i* = 1, …, *N*, defined over the original input space. The individual solutions *P*^*i*^ are then efficiently *merged* together by approximating the merging problem as a relatively small TSP of size *N* + 1, which can be efficiently tackled using a standard solver. We proceed by first considering the end-points $P_{s}^{i},P_{e}^{i}$ of each sub-problem solution *P*^*i*^, and by adding a dummy point connected with zero cost to the start point to have a tour. Each pair of end-points defines an edge $(P_{s}^{i},P_{e}^{i}),\forall i=1,\ldots N$, with a cost equal to the cost of the path between the two end points.
  To effectively merge the individual solution paths, we need to find the path of minimum cost that includes all the edges $(P_{s}^{i},P_{e}^{i}),i=1,\ldots N$ once and only once. This problem is indeed an instance of the *Rural Postman Problem* (RPP), which is unfortunately NP-hard [49]. We tackle it by using a custom heuristic that approximates the the RPP to a TSP and relies on a *sampling* approach to mitigate the effects of the approximation. In general, any RPP-specific heuristic could be used. The advantage of our approach stands on its simplicity and the reuse of standard TSP solvers.
  The final result of the stage of processing consists in one single path, *P*, which represents the incumbent candidate solution for the original OP, and that includes all the nodes in the union set of the individual paths, $\left\{P\right\}={⋃}_{i=1}^{N}\left\{P^{i}\right\}$.
5. **Improve current OP solution**. After the merging, we have one single path *P*, which however is not necessarily optimized since the single path has been composed by merging individual paths. Therefore, the path gets possibly *improved* by running a fast *local search* procedure over the solution set {*P*}.
6. **Repair current OP solution to ensure budget feasibility**. The solution path *P* constructed so far has been built by taking multiple, independently constructed paths *P*^*i*^, *i* = 1, …, *N* and merging them. During the entire process, the cost budget *B* has been explicitly taken into account by letting each sub-problem’s *S*_*i*_, *i* = 1, …, *N*, have a cost budget less than $\frac{B}{N}$, such that the sum of the traveling costs would not exceed the total budget *B*. However, the addition of the edges for the pairwise merging of the sub-paths *P*^*i*^ can make the total traveling cost exceed *B*. At this aim, the path *P* is *repaired* to ensure the satisfaction of the budget constraint. A *greedy heuristic* is used for the purpose of repairing *P*: first, nodes are sorted based on the ratio between the net change in path cost and the net change in path quality that would result from the nodes’ removal from *P*. Then, starting from the end of that sorted list, nodes are removed from *P* until a feasible solution is obtained.
  The resulting path *P* is a feasible, optimized solution for the original OP.

### 3.1 Computing the number of clusters for decomposing the input OP

The core step of the metaheuristic is the partitioning of the *n* input data points into *N* disjoint data sets, hereafter referred to as *clusters* since we use a clustering approach for the purpose of partitioning the input data into disjoint data sets. We will define *balanced* clusters, in the sense that they will include a comparatively equal amount of points $N_{max}\approx \frac{n}{N}$. Based on this operational assumption, *N* is computed as a function of *N*_*max*_ such that the expected time that it would take for the OPS to solve an OP with *N*_*max*_ points is bounded by a time *t*_*cluster*_. In turn, *t*_*cluster*_ is strategically defined as an “acceptable” time in the context of the application (e.g., a computation time of 20 seconds could be acceptable for an interval-based rolling horizon replanning for a mobile robot in a long-lasting mission).

In other words, the value *N*_*max*_ reflects the cost-efficiency of the OPS that we use to solve the OPs of individual clusters, where the use of more efficient solvers allows for a larger *N*_*max*_. Additionally, a larger *N*_*max*_ is expected to result in higher-quality solutions for the global OP, since the possibly negative effects of decomposing the problem into independent sub-problems will be less evident.

Note that the definition of *N*_*max*_ requires some rigorous yet *empirical evaluation process*. In fact, to determine *N*_*max*_, it is first necessary to make a strategic choice about the value of the time bound *t*_*cluster*_, which depends on the application scenario. Then, *t*_*cluster*_ is used to make a possibly empirical assessment of the upper bound on the size of an OP (of the same class considered in the application) that the available OPS can solve within the *t*_*cluster*_ time bound. In our experiments we have precisely considered a data set of instances of increasing size and made a statistical evaluation of the maximum size *N*_*max*_ that could, on average, be solved to optimality or near-optimality within the *t*_*cluster*_ time using our machines.

Once *N*_*max*_ has been strategically defined as described above, *N* is set to:

$$\begin{array}{c} N=\lceil \frac{n}{N_{max}}\rceil . \end{array}$$ (1)

### 3.2 Computing the data clusters for each sub-problem

The computed value of *N* is used to decompose the data set of the input OP into *N* disjoint clusters. In principle, any suitable clustering technique could be used for this purpose. The existing literature is quite extensive, with different algorithms having different pros and cons [50]. In very general terms, clustering techniques can be classified as distance-based, density-based, model-based, grid-based, kernel-based, spectral-based, and hierarchical-based. For decomposing the input set of points into partitions, we need to use a method that ensures that points that are close to each other in the data space are more likely to be in the same group. This is because we need to minimize the distance cost between members of the same group in order to reduce the costs for traversing between consecutive visited sites in the final solution. This would generally allow us to find solutions with more visited sites, which are expected to have higher utility scores. Suitable algorithms for this purpose are distance-based clustering techniques, such as K-means [39] and k-medians [41], that can also run extremely fast and can be strategically tuned in terms of computation vs. accuracy balance. We eventually opted for *K-medians clustering* to partition the input data points because it is more stable and less sensitive to outliers compared to K-means.

The result of the clustering step is that the data of the input OP is split into *N* disjoint sets, *C*_*i*_, *i* = 1, …, *N*, each associated with an edge-weighted graph *G*_*i*_(*V*_*i*_, *E*_*i*_) ⊂ *G*(*V*, *E*) with utilities on the nodes. In turn, each *G*_*i*_(*V*_*i*_, *E*_*i*_) defines a novel, small(er) orienteering sub-problem, *S*_*i*_, *i* = 1, …, *N*.

### 3.3 Solving the OPs inside the *N* clusters

The next stage consists of *solving* the *S*_*i*_, *i* = 1, …, *N* as *independent sub-problems*. The chosen OPS is used for that purpose. Modern multi-core architectures can be readily used for performing the computations in *parallel*, exploiting the full independence among the sub-problems.

Each *S*_*i*_ is defined by its associated graph *G*_*i*_(*V*_*i*_, *E*_*i*_) but also requires a *cost budget constraint* *B*_*i*_. In the input OP, we are given a cost budget *B* for the overall problem, which we can use to define a way of apportioning *B* over the individual sub-problems. The budget *B*_*i*_ allocated for a data cluster *C*_*i*_ should be proportional to the expected utility score that can be gained from that cluster, in order to allow us to spend more time (i.e., visit more sites) in a potentially highly rewarding cluster of points compared to a low rewarding one.

We compute the proportionality factor as follows. We first assign to each cluster *C*_*i*_, *i* = 1, …, *N* a utility score

$$\begin{array}{c} \sigma_{i}=\left|C_{i}\right|U_{i}, \end{array}$$ (2)

where |*C*_*i*_| is the number of points in cluster *C*_*i*_ and *U*_*i*_ is the *median utility score* of points in the cluster. The normalized value of the utility score of a cluster is used to define the fraction of budget allocated for the cluster:

$$\begin{array}{c} B_{i}=\frac{\sigma_{i}}{\sum_{j=0}^{N}\sigma_{j}}B. \end{array}$$ (3)

Once each graph *G*_*i*_ is paired with a budget constraint *B*_*i*_, it is only necessary to add *start* and *terminal* nodes to completely define a new orienteering sub-problem, *S*_*i*_, over the data points in cluster *C*_*i*_. As we have already pointed out, in order to fully account for application scenarios in robotics, we assume that the input OP is a *rooted one*, with the starting node corresponding, for instance, to the robot’s starting location, but the terminal node is open. Therefore, the *S*_*i*_ which includes the root node is defined as a rooted orienteering sub-problem, while all the other orienteering sub-problems are unrooted. In this latter case, a *dummy node* is added to play the role of starting and terminal node. The cost from/to the dummy node is set to zero, as well as the utility delivered by the node.

Each one of the *N* orienteering sub-problems *S*_*i*_ that are now fully defined is *solved* (to optimality or near optimality) using the OPS. Each *S*_*i*_ is tackled independently from the others, allowing to inherently parallelize the computations. In turn, this approach reduces the processing time for solving the input OP to little more than the time to solve the orienteering sub-problem *S*_*i*_ that happens to require the longest processing time.

The output from solving the *N* sub-problems *S*_*i*_ is a set

$$\begin{array}{c} P_{clusters}=\left\{P^{1},P^{2},\ldots ,P^{N}\right\} \end{array}$$ (4)

of *N* mutually disjoint paths, each resulting from the independent solution of a sub-problem *S*_*i*_ defined over a data cluster *C*_*i*_.

### 3.4 Merging solutions from individual orienteering sub-problems

The next stage of the metaheuristic is about *merging the* *N* *disjoint paths in* *P*_*clusters*_ to obtain one single path which will represent a first, not necessarily feasible, solution to the original OP. We merge the paths in a way which is expected to be efficient, aiming to a successive optimization stage to improve the resulting path and make it feasible (if necessary). More specifically, we represent the problem of joining the *N* path solutions in *P*_*clusters*_ first as a *Rural Postman Problem* [49] (RPP) and then we approximate the RPP as an asymmetric TSP using a *sampling* approach.

To model the problem as an RPP, we define the graph *G*_*merge*_ = (*V*_*P*_, *E*_*P*_), where the *vertex* set *V*_*P*_ is the set of all the end-points of the paths *P*^*i*^, *i* = 1, …, *N*, in *P*_*clusters*_:

$$\begin{array}{c} V_{P}=\left\{P_{s}^{1},P_{e}^{1},P_{s}^{2},P_{e}^{2},\ldots ,P_{s}^{N},P_{e}^{N}\right\}, \end{array}$$ (5)

where $P_{s}^{i}$ and $P_{e}^{i}$ represent, respectively, the start and the end nodes of path *P*^*i*^.

The *edge set* in *E*_*P*_ comprises of two subsets: *E*_*P*_ = *E*_*C*_ ⋃ *E*_*NC*_. The set *E*_*C*_ includes all the undirected edges $\left(P_{s}^{i},P_{e}^{i}\right)$ joining the end-points of path *P*^*i*^ inside cluster *C*_*i*_, for *i* = 1, …, *N*. Each edge $\left(P_{s}^{i},P_{e}^{i}\right)$ has a a cost equal to the cost *c*^*i*^ of the path *P*^*i*^ as found in the previous stage.

The set *E*_*NC*_ includes all the undirected edges of type $\left(P_{x}^{i},P_{y}^{j}\right)$ with *x*, *y* ∈ {*s*, *e*} and *i* ≠ *j*, joining the end-nodes of different cluster paths. If the edge $\left(P_{x}^{i},P_{y}^{j}\right)$ was in the initial graph *G*, its cost is set accordingly. Instead, if in *G* the two nodes were not the end-points of an edge, the cost is computed as the shortest path in *G* between the nodes. We have made the assumption that *G* is a connected graph, such that the existence of a shortest path is guaranteed. For instance, in the case of a robotic application where the nodes represents sampling points and the robot can move over continuous environment, the shortest path can be computed as the traveling distance between the two points based on the traversability of the environment and robot’s properties of holonomicity or not.

The goal is to find in the graph *G*_*merge*_ the path of minimum cost that joins all the edges in the subset *E*_*C*_, beginning from the edge with the start node. This defines an instance of the RPP. The RPP is an *arc-routing problem* [49, 51] derived from the Eulerian path problem by requiring to find a minimum cost path through a subset of the edges in the graph. The RPP is NP-hard [49]. Here we tackle it with an original sampling heuristic that allows a direct control of the running times and only requires the use of standard tools for the TSP. The heuristic proceeds as follows.

**Fig 2:**
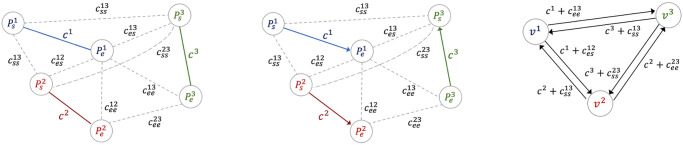
Transformation from RPP to TSP for path merging. (Left) The *G*_*merge*_ graph derived from the solution of the individual *N* sub-problems in *P*_*clusters*_. The costs of associated to each edge are indicated. The undirected cluster edges $\left(P_{s}^{i},P_{e}^{i}\right)$ are represented as solid lines, and different clusters are shown using different colors. (Middle) The *G*_*merge*_ graph with a random selection of the directions for the undirected cluster edges. (Right) The dual transformed graph *G*_*mergeTSP*_, where the cluster edges $\left(P_{s}^{i},P_{e}^{i}\right)$ have been replaced by vertices *v*^*i*^ and the costs of the new edges account for the selected directions of the cluster edges.

We first *transform the RPP problem into an asymmetric TSP*. For each edge $\left(P_{s}^{i},P_{e}^{i}\right)$ in *E*_*C*_ a *direction* is (randomly) selected, either from *s* to *e* or vice versa. Adopting a dual representation, the directed edge is converted into a vertex and the dual graph *G*_*mergeTSP*_ is constructed from *G*_*merge*_ as follows. Each edge $\left(P_{s}^{i},P_{e}^{i}\right)$ in *E*_*C*_ is replaced by a vertex *v*^*i*^. The edges that were before connected to either $P_{s}^{i}$ or $P_{e}^{i}$ become incident to *v*^*i*^ and their cost depends on the selected direction of the original edge $\left(P_{s}^{i},P_{e}^{i}\right)$. The process is illustrated in Fig 2, which shows, from left to right: the *G*_*merge*_ graph, with the costs of associated to each edge, where the undirected cluster edges $\left(P_{s}^{i},P_{e}^{i}\right)$ (i.e., the edges representing the path inside a cluster) are represented as solid lines; a random selection of the directions for the undirected cluster edges; the dual transformed graph *G*_*mergeTSP*_, where the cluster edges $\left(P_{s}^{i},P_{e}^{i}\right)$ have been replaced by vertices *v*^*i*^ and the costs of the new edges account for the selected directions of the cluster edges.

For instance, based on the selected directions shown in the figure, the cost of the edge going from dual vertex *v*^3^ to *v*^1^ includes the cost for traveling from $P_{e}^{3}$ to $P_{s}^{3}$ plus the cost for going from $P_{s}^{3}$ to $P_{s}^{1}$.

The graph *G*_*mergeTSP*_ has now only *N* = 3 nodes and can be solved as an asymmetric TSP, which will be equivalent to find the target minimum cost path joining the *N* cluster edges. To remove the need for a tour and to ensure that the start point is selected as the beginning of the path, a dummy node with zero cost to/from all the other nodes is added to the *G*_*mergeTSP*_ graph and used to define a TSP rooted in start. A merging path can be then found accordingly using a standard TSP solver. We have used the popular *Concorde* https://www.math.uwaterloo.ca/tsp/concorde.html solver (by first transforming the asymmetric TSP into a symmetric one by adding nodes [52]).

Note that the number of clusters may barely go up to an order of 100 in practice. For instance, if the initial OP has size *n* = 10000, which is very large, and the OPS can effectively handle in a few seconds the solution of OPs with 100 nodes, we will have $N=\frac{10000}{100}=100$. Modern TSP solvers, such as Concorde, can tackle TSPs of this size in just a few seconds and output an optimal or near optimal solution.

The TSP approximation of the original RPP is based on choosing arbitrary directions for the cluster edges $\left(P_{s}^{i},P_{e}^{i}\right)$. In order to make an optimized selection among the multiple possible choices of edge directions, we repeat the above process by *randomly sampling* different edge directions. At the end of the iterations, the solution with the cheapest cost is selected. In practice, in our experiments, we stop the sampling loop after a maximum time is reached, which allows a fine control of how much time is being allotted to this processing stage.merg.

### 3.5 Optimize and repair the solution for budget feasibility

The solution path *OP*_*merge*_ found at the previous stage is potentially neither finely optimized nor budget-feasible. Being not finely optimized is the result of having first independently solved a set of sub-problems *S*_*i*_, *i* = 1, …, *N* over disjoint sets of data clusters, and then merged the resulting data points in the *N* paths *P*^*i*^ by only considering the end-points of the paths, and not the whole set of their points.

**Fig 3:**
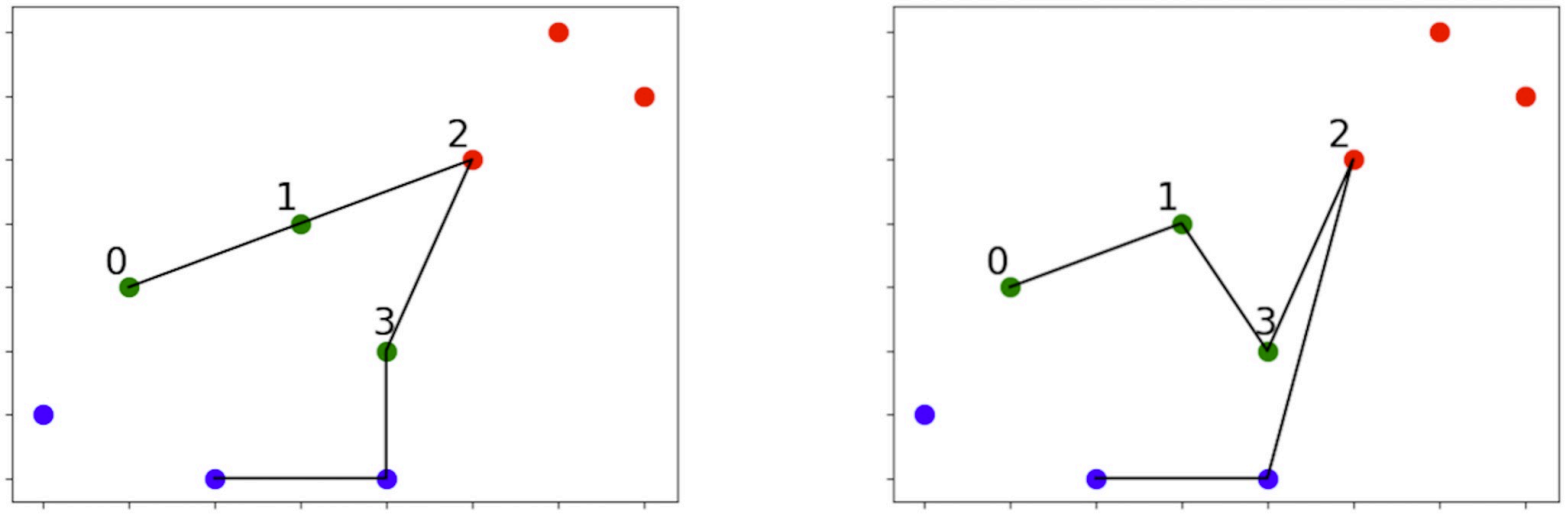
Pitfalls of merging only considering end-points. (Left) Optimal solution of a 9-sites OP. (Right) Solution to the same OP obtained by first decomposing the point set into three clusters. The clusters are highlighted by the different color points. The solution path is the result of merging the solutions from the three clusters using their path end-points.

A simple example of the possible negative effect of the above way of proceeding is shown in Fig 3. In the left figure it is shown the optimal solution path of a simple 9-sites OP. The right figure shows the solution obtained to the same problem through the merging of the solution paths resulting from decomposing the data set in three clusters, highlighted by different colors. The points in the solution are the same but it can be noted that the (short) edge from point 1 to point 2 could not be chosen, since each cluster is solved independently and those two points are in different clusters and one of them is not an end-point. As such, the found solution locally differs from the optimal one, determining a substantial increase in cost, which in turn might make the whole solution not budget-feasible.

In order to tackle the optimization of local components of the solution path *OP*_*merge*_, a *local search optimization procedure* is executed, which outputs a path *OP*_*LS*_ with a possibly lower cost but same total reward compared to *OP*_*merge*_. In our experiments we have chosen a *2-opt local search* for that purpose, given that for the *OP*_*merge*_ we only aim to possibly change the order in which the sites are visited for the sake of reducing the total traveling cost of the solution, without adding or removing sites. In other words, we are dealing with the optimization of a TSP solution, and 2-opt is a fast and effective local search heuristics for TSP [53] that can be easily tuned to run within given strategic time bounds. In any case, the open design of the metaheuristic makes it possible to adopt different choices.

The solution path *OP*_*LS*_ resulting from the local search optimization stage can still be *unfeasible with regards to the cost budget E*. This is because, based on Eq (3), $\sum_{i=1}^{N}B_{i}=B$, we have given no allowance for the cost of inter-cluster traveling, a cost that is added at the time of merging the solutions from solving inside each cluster. This means that there is a concrete possibility that the merged and optimized solution *OP*_*LS*_ has a total cost that is greater than the problem’s budget *B*. We tackle this potential issue by running a *greedy repair procedure* that ensures respecting the budget constraint.

The repair procedure proceeds as follows. First, the nodes in *OP*_*LS*_ are sorted based on a *deletion score* *ds*, computed as the ratio between the cost that would be saved if the node is removed and the utility that would be lost if that node was removed. More precisely, the deletion score for a node *i* ∈ *OP*_*LS*_ is computed as follows, given that in the solution path the node is preceded by a node *k* and followed by a node *j*:

$$\begin{array}{c} ds=\frac{\left(c\left(k,i\right)+c\left(i,j\right)\right)-c_{sp}\left(k,j\right)}{u(i)}, \end{array}$$ (6)

where *c*(*x*, *y*) indicates the cost for crossing the edge between nodes *x* and *y*, *u*(*i*) is the utility provided by node *i*, and *c*_*sp*_ is the cost for going from *k* to *j* along the path of minimal cost (which corresponds to *c*(*k*, *j*) if the two nodes are directly connected). Using the sorted list, the node with the maximum deletion score *ds* is removed from the solution path. This greedy process is repeated until the cost of the overall solution path is less then or equal to the cost budget *B*. Note that, in order to make the score ratio balanced between costs and utilities, both costs and utilities are normalized in a common [0, 1] range.

After the repair stage, the resulting path, *OP*_*feasible*_, is ensured to be a *feasible solution for the original OP*.

The multiple stages of the metaheuristic are summarized below in the pseudo-code of Algorithm 1. Note that for the sake of generality, the algorithms for data clustering and for local search are passed as parameters. In our implementation they have been set, respectively, to K-medians and 2-Opt.

**Algorithm 1**: OP Clustering metaheuristic

**Input**: Orienteering problem (OP), where *P* is the set of candidate points and B is cost budget;

      *OPS*: OP solver;

      *N*_*max*_: Maximum size of a cluster;

      *CA*: Clustering algorithm;

      *LS*: Local search optimizer for merging TSP.

**Output**: Solution path for the input OP.

$k\leftarrow \left\lceil \frac{\left|P\right|}{N_{max}}\right\rceil$ /* *k* is the number of data clusters */

 *S* ← *clusterInputData*(*P*, *k*, *B*, *N*_*max*_, *CA*)

 /* *S* is a set of *k* orienteering sub-problems *S*_*j*_, where each *S*_*j*_ ∈ *S* has at most *N*_*max*_ candidate points */

 **for** *S*_*j*_ in *S* **do**

  *P*^*j*^ ← *OPS*(*S*_*j*_)

  /* Solve all sub-problems using OPS */

  /* If #CPUs > 1, sub-problems can be solved in parallel */

 *P*′ ← *mergePathsWithTSPModel*({*P*^0^, …, *P*^*k*−1^})

 *P*″ ← *improvePath*(*P*′, *LS*)

 **if** *pathIsFeasible*(*P*″, *B*) **then**

  *solutionPath* ← *P*″

 **else**

  *solutionPath* ← *repairPath*(*P*″, *B*)

 **return** *solutionPath*

## 4 Experimental validation using two different solvers as OPS

In the previous section, we have described the multiple stages of the clustering-based metaheuristic. At certain stages, we have made some specific choices, namely the use of K-medians and 2-opt (which we imported from the Concorde TSP solver). Different choices can be made, as long as the objectives of the stage are strategically achieved. The most critical design choice is indeed related to the OPS, the algorithm which is used to tackle the orienteering sub-problems arising from the decomposition by clustering. In this section, we precisely study the impact of using different OPS algorithms. In particular, we consider two extreme approaches, one *evolutionary heuristic*, *EA4OP* [19] and the very popular, *exact solver* *Gurobi* [20]. In their respective domains they can be both regarded as state-of-the-art solutions for OPs.

The EA4OP is a fast and effective heuristic, and in the reference work has been shown to be able to handle relatively large OP instances, with one thousand and more data points. However, computation time grows quite quickly and reaches values that make EA4OP unsuitable, for instance, to use in an online setting. We will show that once plugged in the proposed multi-stage metaheuristic, computation times for the large instances drop of one order of magnitude at the expenses of a very little loss in the solution quality. Note that for making the experiments we have used the code made available by the authors in a Git repository: https://github.com/bcamath-ds/compass.

On the side of Gurobi, it is well understood that in the case of large OPs both the exact solution and a solution with a small MIP gap are out of reach within “acceptable” time bounds. Nevertheless, we will show that, once plugged into our metaheuristic, Gurobi can still be used as a reference solver to tackle large instances within reasonable computation times and good solution quality. This is indeed an interesting option in practice, since Gurobi is a commercial optimization software which is readily accessible for the end-user, while, for instance, installing and using specialized heuristics developed from research groups can be definitely more cumbersome.

As *test instances* we have used instances from the TSPLIB that have been used in the EA4OP paper [19], as well as in other works on orienteering heuristics. Instance sizes range from 101 to 2392 points. Since TSPLIB instances are designed for TSP, node utilities are not explicitly included in the data sets. We have therefore added utilities according to the same two modalities adopted in [19]: (1) all candidate points have the same utility, equal to 1; (2) candidate points have pseudo-random utility scores in the range [1, 100]. In this way we have generated two sets of test instances, that, respectively, we refer to as *Flat* and *Random*.

In the tables below we show the results of running EA4OP and Gurobi over the two sets of instances. For both the algorithms, performance results are shown for the case when the algorithm is used as a standalone solver and when it is plugged into our metaheuristic, playing the role of OPS. The reported values refer to five runs per instance. For the sake of uniformity of comparisons, we have fixed the maximum number of data points per cluster, *N*_*max*_, to 25 for all the experiments. This is indeed a quite low number, especially for EA4OP, and makes the clustering decomposition quite fine grained. Note that in this way we have set challenging conditions for our metaheuristic. The good results that we have obtained are therefore even more valuable.

Note that when using Gurobi we had to set a maximum computation time of 2h and a target MIP gap of 5% in order to keep the computations within acceptable time bounds.

In the tables, the meaning of the columns is as follows (accounting for the multiple runs over the same instance):

- *U*_*EA*_: Utility score using EA4OP, reported as a triple *U*_*max*_, *U*_*mean*_, *U*_*median*_;
- *T*_*EA*_: Computation time in seconds of EA4OP, reported as a pair *T*_*mean*_, *T*_*median*_;
- *U*_*CMH*_: Utility score using clustering metaheuristic, reported as a triple *U*_*max*_, *U*_*mean*_, *U*_*median*_;
- *T*_*CMH*_: Computation time in seconds of clustering metaheuristic, reported as a pair *T*_*mean*_, *T*_*median*_;
- *B*: Cost budget (maximum allowed traveling distance);
- *U*_*G*_: best utility score obtained after optimizing for a maximum of 2 hours (7200 seconds) or when Gurobi raises a memory-related error, MIP gap set to 5%;
- *T*_*G*_: Computation time in seconds of Gurobi exact solver;
- *U*_*CMH*_: Utility score obtained using clustering metaheuristic with Gurobi solver, reported as a triple *U*_*max*_, *U*_*mean*_, *U*_*median*_;
- *T*_*CMH*_: Computation time in seconds of clustering metaheuristic with Gurobi solver, reported as a pair *T*_*mean*_, *T*_*median*_;
- Δ*T*: The relative difference in median computation times between our metaheuristic and the standalone algorithm computed as
  $$\begin{array}{c} \Delta T=\frac{T_{standalone}-T_{meta}}{T_{standalone}}, \end{array}$$ (7)
  where *T*_*standalone*_ is the median computation time of the standalone algorithm and *T*_*meta*_ is the median computation time of our metaheuristic used with that algorithm;
- Δ*U*: The relative difference in median utility scores between our metaheuristic and the standalone algorithm computed as
  $$\begin{array}{c} \Delta U=\frac{U_{standalone}-U_{meta}}{U_{standalone}}, \end{array}$$ (8)
  where *U*_*standalone*_ is the median computation time of the standalone algorithm, *U*_*meta*_ is the median computation time of the metaheuristic with that algorithm.

Note that the experimental results can be *replicated by using the python code that has been provided as supporting information to the paper*.

### 4.1 Clustering metaheuristic with EA4OP as heuristic solver

Tables 1 and 2 clearly show that our metaheuristic is capable of sharply attenuating the computation time of the standalone EA4OP algorithm, hereafter referred to as *H*_*EA*4*OP*_, as the size of the OP increases. For smaller instances, which are not our primary focus, *H*_*EA*4*OP*_ might be able to compute solutions in marginally less computation times than when it is plugged into our metaheuristic, which we refer to as *MH*_*EA*4*OP*_. As the instance size exceeds 300, the gain in computation time achieved by *MH*_*EA*4*OP*_ over *H*_*EA*4*OP*_ becomes substantial. In fact, the larger the OP instance, the greater the reduction in computation time achieved by *MH*_*EA*4*OP*_, reaching as much as a 95% relative reduction in computation time.

**Table 1:** Performance of EA4OP as standalone algorithm and when used in the clustering metaheuristic (*Flat* instances).

Instance	*B*	*U*_*EA*_	*T*_*EA*_	*U*_*CMH*_	*T*_*CMH*_	Δ*T*	Δ*U*
eil101	315	64, 64, 64	0.6, 0.6	60, 58, 58	1.3, 1.6	-1.67	0.09
gil262	1189	156, 155, 154	3.4, 3.5	130, 130, 130	3.5, 2.7	0.23	0.16
pr299	24096	158, 158, 158	2.8, 2.8	146, 143, 142	1.3, 1.3	0.54	0.10
lin318	21015	201, 198, 200	5.9, 4.8	182, 176, 176	3.0, 1.8	0.63	0.12
rd400	7641	235, 233, 233	6.59, 6.6	195, 191, 192	1.6, 1.6	0.76	0.18
d493	17501	314, 312, 312	16.8, 16.5	301, 292, 289	4.7, 3.1	0.81	0.07
u574	18453	339, 335, 334	16.3, 15.6	311, 301, 301	5.2, 3.7	0.76	0.10
u724	20955	420, 418, 419	25.6, 25.2	386, 377, 375	5.9, 3.7	0.85	0.11
pcb1173	28446	636, 626, 627	61.0, 61.4	574, 565, 565	7.4, 7.7	0.87	0.10
fl1400	10064	1039, 1037, 1038	535.7, 558.8	888, 825, 875	29.5, 27.3	0.95	0.16
pr2392	189016	1282, 1238, 1226	371.0, 392.7	1156, 1141, 1137	24.8, 26.7	0.93	0.07

**Table 2:** Performance of EA4OP as standalone algorithm and when used in the clustering metaheuristic (*Random* instances).

Instance	*B*	*U*_*EA*_	*T*_*EA*_	*U*_*CMH*_	*T*_*CMH*_	Δ*T*	Δ*U*
eil101	315	3655, 3646, 3646	0.8, 0.6	3442, 3390, 3365	1.0, -0.7	0.17	0.08
gil262	1189	8105, 8055, 8104	2.6, 2.8	6960, 6845, 6868	4.8, 2.4	0.14	0.15
pr299	24096	9029, 8934, 8953	3.3, 3.2	8473, 8153, 8067	7.5, 6.7	-1.09	0.10
lin318	21015	10777, 10735, 10724	8.5, 7.9	9933, 9485, 9792	2.0, 2.0	0.75	0.09
rd400	7641	13426, 13332, 13318	6.7, 6.3	11431, 11374, 11427	1.9, 1.8	0.71	0.14
d493	17501	16822, 16790, 16780	20.5, 21.6	15135, 14816, 15134	10.6, 13.5	0.38	0.10
u574	18453	18936, 18899, 18913	15.8, 15.2	16619, 15920, 16014	5.7, 3.7	0.76	0.15
u724	20955	23621, 23454, 23441	32.7, 26.4	20502, 20362, 20338	4.4, 4.3	0.84	0.13
pcb1173	28446	35613, 35240, 35055	60.3, 59.3	31847, 31717, 31784	12.8, 15.5	0.74	0.09
fl1400	10064	56032, 55309, 55242	684.9, 627.6	47389, 44479, 44833	29.8, 29.6	0.95	0.19
pr2392	189016	70023, 69595, 69475	360.4, 368.6	62713, 61505, 61740	32.6, 28.8	0.92	0.11

On the other hand, the relative reduction in solution quality caused by using *MH*_*EA*4*OP*_ instead of *H*_*EA*4*OP*_ does not seem to be correlated, either negatively or positively, with the size of the OP instance. In fact, the reduction in solution quality, which ranges from 7% to 19% in our experiments, remains relatively flat as the size of the problem increases.

Given the difference in how Δ*U* and Δ*T* change with the increase in the OP instance size, it is evident that, the larger the size of the OP instance, the more beneficial it is to use *MH*_*EA*4*OP*_ instead of *H*_*EA*4*OP*_. To demonstrate this more clearly, we assign a score that represents the gain achieved by using *MH*_*EA*4*OP*_ versus *H*_*EA*4*OP*_ to solve an OP instance. We define this *gain score*, *G*, as

$$\begin{array}{c} G=\Delta T-\Delta U. \end{array}$$ (9)

**Fig 4:**
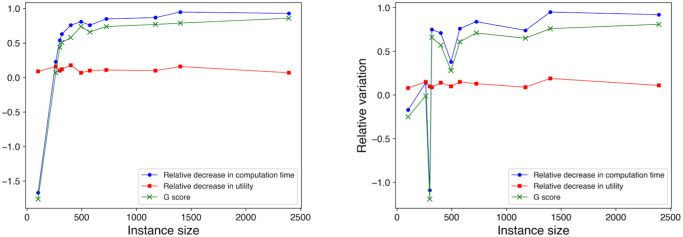
Performance of *MH*_*EA*4*OP*_ vs. *H*_*EA*4*OP*_. Gain achieved by using *MH*_*EA*4*OP*_ instead of *H*_*EA*4*OP*_ vs. instance sizes. (Left) OP instances with *Flat* utility scores. (Right) OP instances with *Random* utility scores.

A positive *G* score means that the decrease in computation time achieved by using *MH*_*EA*4*OP*_ instead of *H*_*EA*4*OP*_ outweighs the decrease in utility, and thus it is “beneficial” to use *MH*_*EA*4*OP*_ instead of *H*_*EA*4*OP*_. Note that a negative value of Δ*T* or Δ*U* indicates a relative increase in computation time or solution utility, respectively, relative to the standalone algorithm. Fig 4 shows how Δ*T*, Δ*U*, and *G* change with the increase of the OP instance size. The two plots indicate that the gain score sharply increases with the increase of problem size. The *G* score then plateaus as the relative decrease in computation time approaches 100%, given that the relative decrease in utility score remains relatively flat.

### 4.2 Clustering metaheuristic with the Gurobi exact solver

Tables 3 and 4 show the results of the experiments carried out using the Gurobi exact solver, referred to as *H*_*G*_, and our metaheuristic with the Gurobi solver plugged into it, referred to as *MH*_*G*_. The results in those tables should be examined while keeping in mind that, in the experiments with *H*_*G*_, a maximum time for computation was set to 2 hours (7200 seconds). Additionally, a MIP gap goal was set to 0.05 (5%). This means that *H*_*G*_ stops computing once 2 hours has passed or once the incumbent solution reaches a MIP gap of 0.05. When using the Gurobi solver inside *MH*_*G*_, computations for each cluster were interrupted either after 40 seconds of computation or when the incumbent solution inside the cluster reached a MIP gap of 0.05. Given these settings, we would expect that, for large(er) OPs, the Gurobi solver in our experiments would not be able to achieve high utility scores, even after exhausting all of the allowed computation time. As a matter of fact, for the largest instance used in the experiments, pr2392, no feasible solution was found by *H*_*G*_ in the given time bounds. The result of these limitations for computation times is that *MH*_*G*_ achieves better utility scores than *H*_*G*_ for the larger OP instances, which is indicated by the negative values in the Δ*U* column of the tables.

**Table 3:** Performance of Gurobi as standalone algorithm and when used in the clustering metaheuristic (*Flat* instances).

Instance	*B*	*U*_*G*_	*T*_*G*_	*U*_*CMH*_	*T*_*CMH*_	Δ*T*	Δ*U*
eil101	315	62	44.7	60, 59, 59	1.3, 1.3	0.97	0.05
gil262	1189	148	7200	139, 131, 129	22.7, 22.9	0.99	0.13
pr299	24096	162	7200	143, 139, 140	36.8, 39.4	0.99	0.14
lin318	21015	152	7200	191, 189, 189	69.2, 64.9	0.99	-0.24
rd400	7641	217	7200	208, 202, 201	55.0, 65.0	0.99	0.07
d493	17501	211	7200	311, 296, 300	38.4, 34.4	0.99	-0.42
u574	18453	285	7200	295, 293, 295	167.5, 163.1	0.98	-0.04
u724	20955	367	7200	369, 363, 365	86.0, 84.4	0.99	0.01
pcb1173	28446	559	7200	583, 578, 581	48.0, 42.7	0.97	-0.04
fl1400	10064	499	7200	988, 863, 816	334.6, 387.7	0.87	-0.64
pr2392	189016	NA	NA	1159, 1145, 1142	525.2, 557.3	NA	NA

**Table 4:** Performance of Gurobi as standalone algorithm and when used in the clustering metaheuristic (*Random* instances).

Instance	*B*	*U*_*G*_	*T*_*G*_	*U*_*CMH*_	*T*_*CMH*_	Δ*T*	Δ*U*
eil101	315	3558	620	3362, 3316, 3349	4.0, 4.6	0.99	0.06
gil262	1189	8025	7200	7353, 6882, 6686	43.9, 45.7	0.99	0.17
pr299	24096	7954	7200	8062, 7705, 7893	65.3, 65.9	0.99	0.01
lin318	21015	9044	7200	9928, 9727, 9853	135.9, 111.8	0.98	-0.09
rd400	7641	10890	7200	11397, 11314, 11347	74.8, 66.1	0.99	-0.04
d493	17501	14232	7200	15355, 14913, 14755	145.5, 144.7	0.98	-0.04
u574	18453	13738	7200	16506, 16159, 16225	233.9, 229.2	0.97	-0.18
u724	20955	16211	7200	20669, 20589, 20628	112.8, 119.9	0.98	-0.27
pcb1173	28446	21616	7200	31664, 31360, 31272	98.0, 69.8	0.96	-0.45
fl1400	10064	3682	7200	48396, 41512, 45032	557.4, 554.0	0.92	-11.23
pr2392	189016	NA	NA	63570, 63253, 63209	761.6, 768.5	NA	NA

**Fig 5:**
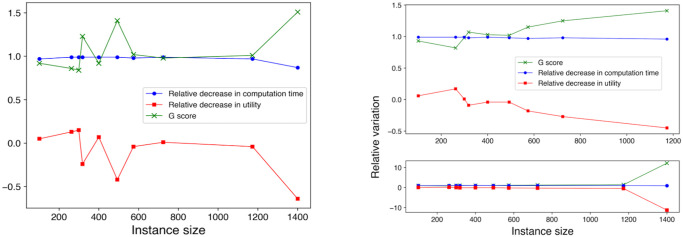
Performance of *MH*_*G*_ vs. *H*_*G*_. Gain achieved by using *MH*_*G*_ instead of *H*_*G*_ vs. instance sizes. (Left) OP instances with *Flat* utility scores. (Right) OP instances with *Random* utility scores. In the upper plot, gains do not include the largest test instance, pr2392, since in this case the relative improvement using *MH*_*G*_ is two orders of magnitude such that data points for the other instances get flattened out. The lower plot is the same as the upper one but including the data for pr2392.

Δ*T* for the Gurobi solver experiments remained very high for all the instances. This is due to the fact that, the less time-efficient the OP solver, the more gain is achieved by decomposing the problem into smaller sub-problems. Since the Gurobi solver can be inefficient for medium-large instances, our metaheuristic is able to achieve a very substantial computation time reduction. The gain score plots for *H*_*G*_ and *MH*_*G*_ are shown in Fig 5. In this case they look more irregular than in the EA4OP case precisely due to the fact that some values become negative.

Note that the code for the experiments reported in this paper is not parallelized. To quantify the effect of solving for the clusters in parallel, we solved the pr2932 instance with EA4OP plugged into our metaheuristic. The number of clusters computed was 96. The computation times for the clusters have an average of 0.15 seconds and a median of 0.16 seconds. To emulate the effect of solving the clusters in parallel, we randomly assigned each cluster to one of 4 CPU cores of our machine. As a result, the computation cost of the 96 clusters went down from a total of 11 seconds to only 3 seconds (72% reduction). If we had more than four cores, the reduction would be more significant, bringing ideally the whole computation time of the metaheuristic to little more the time of computing for one cluster, about 1 second in the example case, which would definitely be suitable for an online deployment.

## 5 Conclusions and future work

In this paper, we have introduced a new metaheuristic for tackling the Orienteering Problem, seeking to improve existing algorithms and allow for their effective use in an online setting. In particular, we have emphasized that this could be a typical scenario when using mobile robotic agents for tasks such as environmental monitoring, surveillance, and goods delivery. The metaheuristic presented in this paper aims to mitigate the typical exponential growth in the computation time of state-of-the-art OP algorithms when faced with large OPs. The metaheuristic implements an efficient decomposition approach to the problem and allows to drastically reduce the used computation time of an OP algorithm by paying a strategically controlled and comparatively small price in terms of quality loss.

The metaheuristic goes through multiple stages of processing and optimization, flexibly allowing the user to customize individual parts in terms of both quality and used computational resources. Given an input OP and an OP algorithm to use (i.e., to improve in terms of final efficiency), the first stage is based on a clustering approach that divides the input orienteering problem into sub-problems whose sizes are bounded by a given parameter. This size parameter can be strategically set to reflect the efficiency of the OP algorithm used inside the metaheuristic. Sub-problems are treated as independent of each other, such that, when multiple CPU cores are available, the sub-problem set can be very efficiently tackled in parallel. In a second stage, solutions for the sub-problems, which represent a path inside each cluster, are joined into one solution using a TSP model that guarantees extremely rapid solution. In successive stages, through fast local optimization and repair procedures, path quality is boosted and its feasibility is ensured.

Based on this general processing architecture, we have carried out an empirical evaluation of the balance between computational efficiency and solution quality that can be obtained using the metaheuristic. In particular, as OP algorithms to possibly “improve”, we have considered two state-of-the-art approaches featuring different characteristics and properties. The experimental results strongly support the effectiveness of using the metaheuristic. For both the algorithms, when plugged in the metaheuristic, solution quality suffers from relatively little or no loss compared to the case when they are used as standalone algorithms. However, at the same time, their computation times get dramatically reduced, potentially paving the road for their use in online scenarios, and, in general, for efficiently tackling large OP instances.

**Future work** will address a few limitations of the current design and implementation of the metaheuristic. In Section 3 we have made the assumption of a connected graph for encoding the OP, where this assumption is aimed at simplifying the merging stage (Section 3.4). Since this assumption may not necessarily be valid in real-world applications, it will be relaxed and efficient and feasible ways of merging will be developed (e.g., by locally adapting the paths inside a cluster). We will also explore the use of different procedures for implementing the improve and repair stage (Section 3.5), aiming to give the user an even better strategic control over the use of computational resources. Still regarding the improvement of computational efficiency, we will perform an actual parallel implementation of the code, to be able to fully exploit the presence of multiple cores. Finally, and maybe more importantly, we will extend our metaheuristic to use it for the Team Orienteering Problem (TOP), possibly including the presence of proximity constraints (e.g., to support networking, as well as to manage rendez-vous between agents). The plain extension to TOPs is rather straightforward, since it only requires adding an initial fine-grained clustering stage to identify clusters to be assigned to each agent, and then proceed as it is now. Instead, dealing with proximity constraints will require the introduction of additional design solutions. Note that the extension to TOPs, as well as the attention toward proximity constraints, is particularly interesting when using orienteering models in multi-robot scenarios, which is one of our driving motivations behind this work.

## Supporting information

S1 Data (ZIP)

S2 Data (ZIP)
